# Progresses and Perspectives of Anti-PD-1/PD-L1 Antibody Therapy in Head and Neck Cancers

**DOI:** 10.3389/fonc.2018.00563

**Published:** 2018-11-28

**Authors:** Bo Yang, Tingjun Liu, Yang Qu, Hangbo Liu, Song Guo Zheng, Bin Cheng, Jianbo Sun

**Affiliations:** ^1^Guanghua School of Stomatology, Hospital of Stomatology, Sun Yat-sen University, Guangzhou, China; ^2^Guangdong Provincial Key Laboratory of Stomatology, Sun Yat-sen University, Guangzhou, China; ^3^Division of Rheumatology, Penn State Health Milton S. Hershey Medical Center, Hershey, PA, United States

**Keywords:** PD-1, PD-L1, immune checkpoint inhibitor, head and neck cancer, immunotherapy, adverse effects

## Abstract

Head and neck cancer is the 6th most common malignancy worldwide and urgently requires novel therapy methods to change the situation of low 5-years survival rate and poor prognosis. Targeted therapy provides more precision, higher efficiency while lower adverse effects than traditional treatments like surgery, radiotherapy, and chemotherapy. Blockade of PD-1 pathway with antibodies against PD-1 or PD-L1 is such a typical targeted therapy which reconstitutes anti-tumor activity of T cell in treatments of cancers, especially those highly expressing PD-L1, including head and neck cancers. There are many clinical trials all over the world and FDA has approved anti-PD-1/PD-L1 drugs for head and neck cancers. However, with the time going, the dark side of this therapy has emerged, including some serious side effects and drug resistance. Novel materials like nanoparticles and combination therapy have been developed to improve the efficacy. At the same time, standards for evaluation of activity and safety are to be established for this new therapy. Here we provide a systematic review with comprehensive depth on the application of anti-PD1/PD-L1 antibodies in head and neck cancer treatment: mechanism, drugs, clinical studies, influencing factors, adverse effects and managements, and the potential future developments.

## Introduction of head and neck cancers

Head and neck cancers are composed of various kinds of epithelial malignant tumors, including oral cancers, maxillofacial cancers, larynx cancers, and many others, almost all of which are head and neck squamous cell carcinoma (HNSCC). Although, there are other pathological types such as verrucous carcinoma, basaloid squamous cell carcinoma, papillary squamous cell carcinoma, they only make up a small percentage (1). HNSCC is the 6th most common malignancy worldwide, with number of 650,000 new cases a year and 350,000 deaths (2). Around 2/3 of patients present with advanced disease, often with regional lymph node involvement, while 10% present with distant metastases (3). According to epidemiological survey, the 5-years survival rate of HNSCC in all stages was about 60%, and the survival rate was even worse for specific primary sites such as hypopharynx. The main causes of head and neck cancers are tobacco and alcohol consumption (1, 4–8). Chewing betel quid is also well-recognized as a risk factor for the cancer of oral cavity (9). And human papillomavirus (HPV) and p53 mutation are related to certain subsets of head and neck cancers (10–12). About 25% of HNSCC contain HPV genomic DNA (13). However, HPV positivity is a favorable prognostic factor in HNSCC (14). Patients with HPV^+^ HNSCC show better responsiveness to radiation, chemotherapy, or both, and might be more susceptive to immunosurveillance of tumor-specific antigens (14).

## Common treatment strategies for head and neck cancers

The location of the cancers makes it necessary to take the spiritual and plastic factors into consideration. Primary tumor site, stage, and resectability are also treatment concerns as well as the patient factors such as swallowing, airway, organ preservation, and comorbid illnesses. For plan making, doctors are needed and organized from different departments which include head and neck surgeons, plastic surgeons, medical oncologists, radiation oncologists, radiologists, and dentists (2).

Common treatment strategies for head and neck cancers include surgery, radiotherapy, and chemotherapy. At present, surgery is still the standard therapy for HNSCC. However, surgical operations are limited, owing to the complexity of structures and the need for organ preservation. Most surgeons agree that the carotid artery, the base of the skull, and the invasion of the pre-vertebral muscle tissue are unresectable (2). Moreover, when the tumor is too extensive or there are multiple distant metastases, patients are generally not suitable for surgical treatment. Radiotherapy alone can improve the cure rate of early glottis, tongue, and tonsil cancers (15). However, prolonged interruption of radiotherapy or delayed post-operative radiotherapy may impair the patient's prognosis, which may be due to the proliferation of cancer cells (16). Delivery of radiation remains to be improved with continuous technological progress, and customization of radiation dose and volume (17). Chemotherapy is the core component of local advanced HNSCC treatment (18). Platinum compounds Cisplatin is a standard reagent for combination with radiotherapy or other drugs. Huperzine compounds are active and have been tested in locally advanced HNSCC chemotherapy (19, 20). Concurrent chemotherapy with normo-fractionated radiotherapy (2 Gy/day, 5 days/week, for 5–7 weeks) is used most in current practice (21).

Traditional therapy can result in serious complications, from pain to malnutrition, risk of infection, and psychological distress (21). In order to ameliorate these drawbacks, comprehensive treatments are currently preferred for the advanced tumors. Comprehensive treatments must be well-designed and planned according to the patient's general condition and the stage of tumor development. At present, the treatment of oral and maxillofacial malignant tumors emphasizes the comprehensive treatment based on surgery, especially the triple therapy, which combines surgery with radiotherapy and chemotherapy.

**Figure 1:**
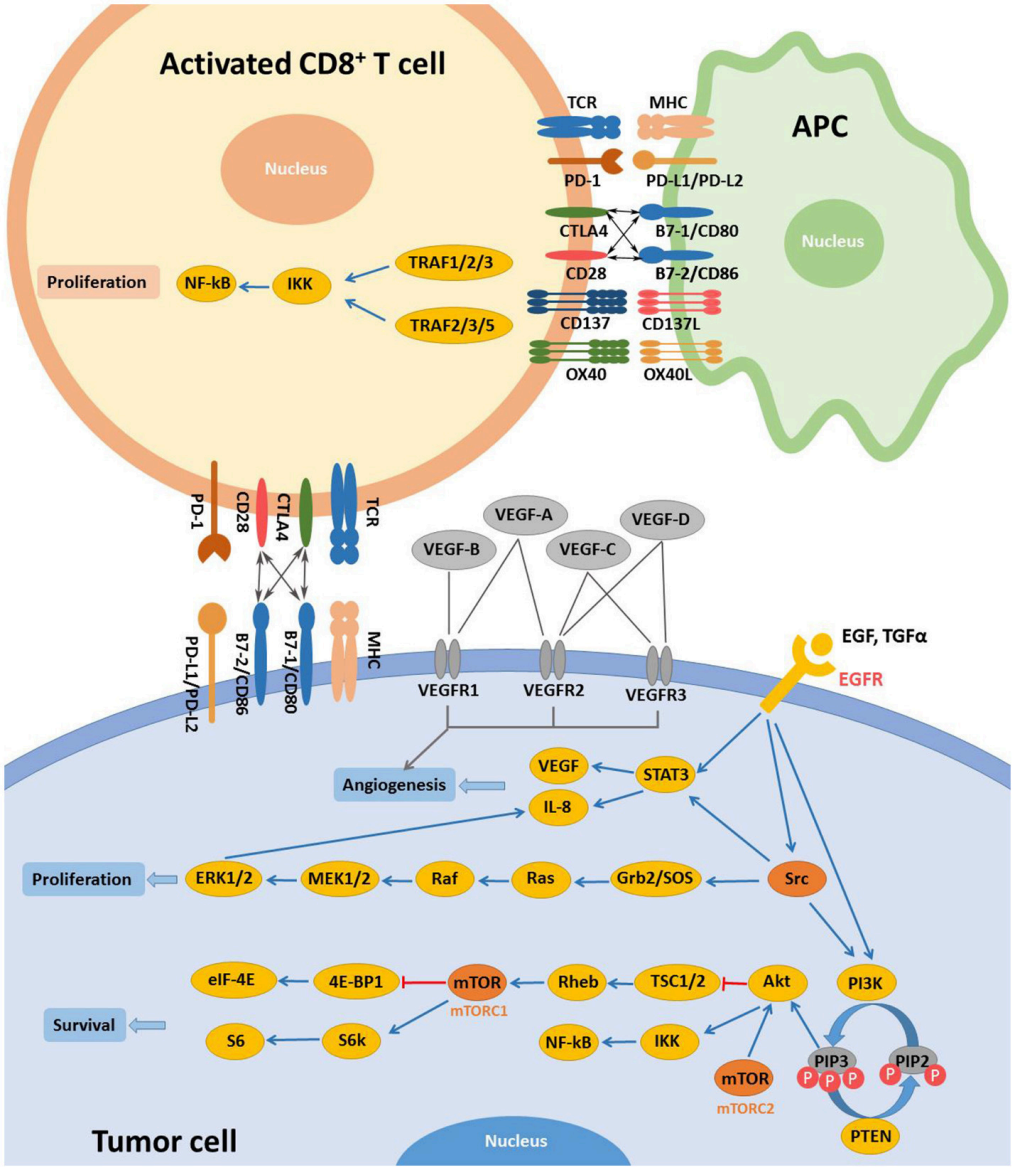
Main targets and related signaling pathways involved in the targeted therapy for R/M HNSCC. Activation of EGFR by extracellular ligands initiates activation of Src, STAT3, and PI3K. Activated Src promotes cell proliferation mainly via RAS/RAF/MAPK pathway. In the PI3K/Akt pathway, phosphorylation of PIP2 is mediated by PI3K while dephosphorylation of PIP3 is controlled by PTEN. Akt could be activated independently by mTORC2 activation. Activation of Akt and mTORC1 inhibit TSC1/2/Rheb and 4E-BP1/eIF-4E downstream signaling, respectively while IKK/NF-kB and S6/S6k pathways are initiated, promoting tumor cell survival. Once activated, other targets, including VEGFR and c-MET, expressed on tumor cells share similar downstream signaling with EGFR. CD137L and OX40L activate CD137 and OX40, respectively. And proliferation of activated T cells is achieved via TRAF/IKK/NF-κB downstream signaling. CTLA-4 and its ligands are also demonstrated. Some pathways were simplified for clearer demonstration.

Modern research has been keen on identifying specific molecular targets involved in the occurrence and progression of head and neck cancers. EGFR and VEGF are two main targets which are overexpressed in majority of both precancerous oral lesions and HNSCC (22–24). EGFR can bind to and be activated by different ligands, including the epidermal growth factor (EGF) and transforming growth factor-α (TGF-α) (25). EGFR activation initiates subsequent signaling pathways, eventually resulting in tumor cell resistance to apoptosis and promoting angiogenesis, tumor cell migration, and tumor cell proliferation (Figure 1) (25, 26). Current EGFR-targeted therapies include monoclonal antibodies (mAbs) and tyrosine kinase inhibitors (TKIs). Antibodies target the extracellular domain of EGFR while TKIs hinder downstream signaling pathways by binding to the cytoplasmic region of EGFR (27). To date, Cetuximab remains the only FDA-approved EGFR-targeted mAb for the treatment of recurrent/metastatic (R/M) HNSCC. Cetuximab in combination with radiotherapy is a standard treatment option for locally or regionally advanced HNSCC (28). VEGF, is a key regulator of physiological angiogenesis during embryogenesis, skeletal growth, and reproductive functions (29). The biological effects of VEGF, mediated by two receptor tyrosine kinases (RTKs), VEGFR-1 and VEGFR-2, cause receptor TK activation and downstream signaling to stimulate endothelial cell proliferation, vessel permeability, and migration (27). Bevacizumab, a humanized monoclonal antibody targeting VEGF-A, was approved by the FDA for treatment of advanced cancer types. Bevacizumab could increase the sensitivity of HNSCC to radiotherapy in preclinical trials. Bevacizumab was evaluated in phase I and II clinical trials in combination with Erlotinib, an EGFR inhibitor, in patients with R/M HNSCC (30, 31) and the combined treatments increased the complete response rate by ~15% and median survival by 7.1 months (30). The phase II trial on the combination of Bevacizumab with chemotherapy, radiotherapy or EGFR inhibitors are ongoing.

## Immunological targeted therapy

Immunotherapies stimulate host antitumor immune system and can elicit endurable responses in subsets of patients across different types of tumors (Figure 1) (32). Immune checkpoints, like cytotoxic T-lymphocyte-associated antigen-4 (CTLA-4) and programmed cell death-1 (PD-1), work as inhibitory pathways, playing an important role in self-tolerance under healthy conditions. Checkpoint inhibitors are part of immunotherapies that enhance antitumor T cell activity by hindering initiation of suppressive signaling pathways of activated T cells. The 2018 Nobel Prize in Physiology or Medicine was recently given to James P. Allison and Tasuku Honjo for their discovery and contribution in cancer immunotherapy correlated with CTLA-4 and PD-1. Other targets such as CD137 and OX40, unlike CTLA-4 and PD-1, work as immune activators and are as well under active investigation for cancer therapy (Table 1) (37, 38).

**Table 1:** Immunological targeted therapies approved or under investigation for the treatment of head and neck cancers.

**Drug**	**Target**	**Modality**	**Status**	**References**
MEDI0562	OX40	Antibody	Phase I Phase Ib Phase IPhase I	NCT03336606 NCT02315066
Urelumab	CD137	Antibody	Phase IPhase I	NCT02110082
PF-05082566	CD137	Antibody	Phase IPhase I	NCT02315066
Ipilimumab	CTLA-4	Antibody	Phase II Phase IPhase I Phase IPhase I	NCT03620123 NCT03098160 NCT02812524
Tremelimumab	CTLA-4	Antibody	Phase III Phase III Phase II Phase II Phase IPhase I-2	NCT02369874 NCT02551159 NCT03624231 NCT03292250 NCT03019003
Pembrolizumab	PD-1	Antibody	Approved	(33, 34)
Nivolumab	PD-1	Antibody	Approved	(35, 36)
Darvalumab	PD-L1	Antibody	Phase III Phase II Phase IPhase I	NCT02551159 NCT02207530 NCT02997332
Avelumab	PD-L1	Antibody	Phase III Phase IPhase I	NCT02952586 NCT02938273
INCB024360	PD-L1	Antibody	Phase IPhase I/2	NCT02318277

*PD-1, programmed cell death protein 1; PD-L1, programmed death ligand 1; CTLA-4, cytotoxic T lymphocyte-associated antigen-4*.

### CTLA-4

Cytotoxic T lymphocyte-associated antigen-4 (CTLA-4; also known as CD152) is the first clinically targeted immune checkpoint receptor. CTLA-4, expressed on activated CD8^+^ effector T cells, mainly regulates the early stage of T cell activation, enhances the activity of effector CD4^+^ T cell, and inhibits Treg cell-dependent immunosuppression (39, 40). CD28 and CTLA-4 have the same ligands B7-1 (also known as CD80) and B7-2 (also known as CD86); and CTLA-4, compared to CD28, has a much higher affinity for B7-1 (41). CTLA-4 has been proved to be a negative regulator of T cell activation in an effort to prevent autoimmunity, antagonizing the CD28-B7 co-stimulatory signals. Research showed that the blockade of CTLA-4 results in enhanced antitumor immunity (42). Clinical studies using anti-CTLA-4 antibodies demonstrated activity in melanoma. Ipilimumab, an anti-CTLA-4 antibody, was the first targeted immunotherapy to prove a survival advantage for patients with metastatic melanoma. Hence, it was approved by FDA for the treatment of advanced melanoma in 2010 (43). In HNSCC, Yu et al. showed that CTLA4 was upregulated in the tumor-infiltrating lymphocyte (TIL) of HNSCC and the high CD8^+^/CTLA4 ratio was associated with improved prognosis (44). Further, Jie et al. found that intratumoral Tregs, compared to circulating Tregs, induced higher expression of CTLA-4 in HNSCC (45). Currently, clinical trials of Ipilimumab (NCT02551159, NCT03212469), alone or in combination with other treatments, for HNSCC are in progress (40).

### CD137

CD137, a member of TNF receptor superfamily, is widely induced on activated CD4^+^ T cells, CD8^+^ T cells, B cells, NK cells, monocytes, and DC. The engagement of CD137 could promote the proliferation of T cells. The introduction of Urelumab, the fully human CD137-agonist mAb, has enabled modulation of CD137 function in immune-oncology, including application in combination with tumor targeting mAb (46). Srivastava et al. (38) confirmed that Cetuximab combined with CD137 agonist was effective in the treatment of HNC. CD137 has provided a new mechanism for the enhancement of Cetuximab (38).

### OX40

OX40 is a member of the TNF receptor family and mediates an effective co-stimulation pathway which can enhance T cell memory, proliferation, and antitumor activity in patients with metastatic cancers (47, 48). Overexpression of OX40 in the TIL of patients with HNSCC has been identified (49). Furthermore, Montler et al. have noted co-expression of OX40 with PD-1 and CTLA-4 in a majority of tumor specimens, especially within the Treg population (49). The preclinical model showed the synergistic effects of anti-OX40 and anti-PD1, anti-OX40 and anti-CTLA-4, as well as anti-OX40 and anti-PDL1 (49). Anti-OX40 is currently being tested in early clinical trials of HNSCC, both as monotherapy and in combination with other immunotherapies (37).

## Anti-PD-1/PD-L1 therapy

T cells express the inhibitory receptor known as PD-1 on their surfaces to guard our body (50). When bound by its ligands PD-L1 or PD-L2, PD-1 transduces a signal into T cells to attenuate downstream signaling through the PI3K and PKCθ pathways (50, 51), which results in inhibition of T cell activation and proliferation. This protective mechanism is also utilized by tumor cells to escape immune attack through expressing high abundance of PD-L1 ligands on their surfaces.

Anti-PD-1/PD-L1 therapy has been a routine treatment to patients with PD-L1 highly expressing tumor (52). This kind of immunotherapy could target tumors more precisely. Meanwhile, as anti-PD-1/PD-L1 therapy has been applied to more and more patients, the side effects and the factors hindering the therapeutic effects have been noticed. Thus, combined treatments and better administrating methods have been raised to improve the treatment.

### Mechanism of PD-1/PD-L1 inhibitors

**Figure 2:**
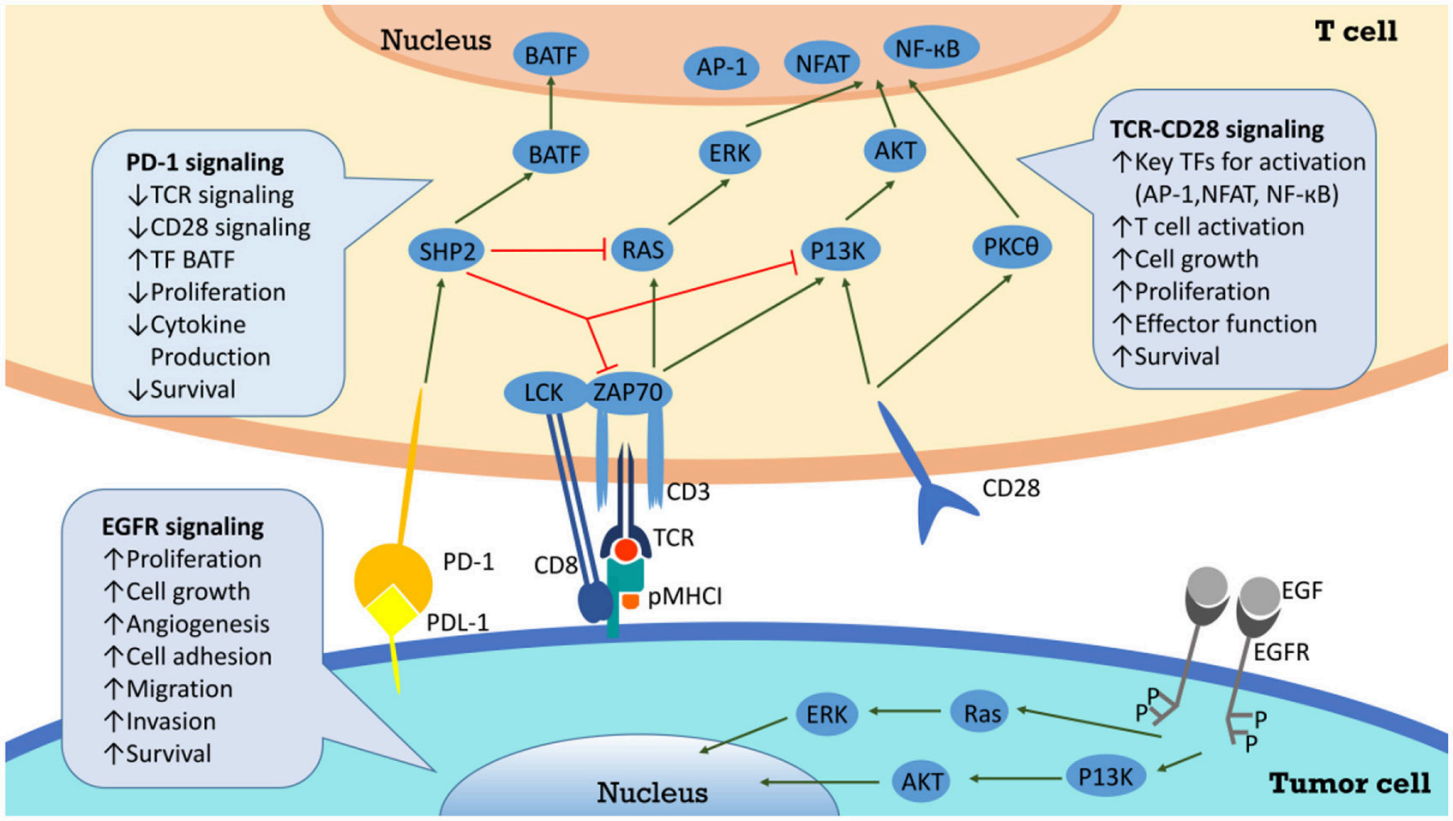
PD-L1/PD-1 signaling pathway and the correlated network. Interaction between PD-L1 and PD-1 on T cells results in inhibition of Zap70 phosphorylation and PI3K activation, and finally attenuates TCR signaling, CD28 mediated co-stimulation, NF-κB, and AP-1 activation, and IL2 production. Through inhibition of T cell via overexpression of PD-L1, cancer cells evade the host immune system.

Tumor infiltrating lymphocytes, especially CD8^+^ T cells, exhibit high levels of PD-1 in HPV^+^ HNSCC (12). When PD-1 binds to PD-L1 on tumor cells, T cell proliferation is suppressed and tumor cells are able to evade immune attack more effectively in the tumor microenvironment (12). Since tumors expressing PD-L1, compared to PD-L1–negative tumors, showed improved response to Nivolumab (a PD-1 inhibitor) (53), it is important to investigate the level of PD-L1 expression in tumor microenvironment. One study suggested that patients with HPV^−^ HNSCC expressed high levels of PD-1 in T cells and PD-L1 in a majority of tumor cells (54). Despite primary tumor sites, PD-L1 has been spotted on metastatic lesions (55). In summary, more than 29% of HPV^−^ and around 70% of HPV^+^ HNSCC express PD-L1, suggesting that the majority of these cancers have potential for responding to PD-1 inhibitors (56). PD-L1 and PD-1 interaction is among the signals beneficial for tumor cells, which also include EGFR signaling, CD 28 stimulation and many others. And there are plenty of downstream pathways as well, which are composed of SHP2, RAS, ZAP70, P13K, and so on (Figure 2).

When bond by PD-1 ligands, PD-1 is able to recruit phosphatases including SHP2 toinhibit T cell functions by countering the positive signaling events mediated by the T cell receptors (TCR) and CD28 (50). For instance, they restrain ZAP70 and PI3K–AKT and RAS signaling pathways (50). In conclusion, this lowers down the activation of transcription factors such as AP-1, NFAT, and NF-κB, which are important for T cell activation, proliferation, growth, and survival. Besides, PD-1 is able to inhibit T cell functions by improving the expression of BATF transcription factor to inhibit the effector transcriptional programs. EGFR is an important target for mediating tumor metastasis and adhesion. After combining with epidermal growth factor (EGF), EGFR can deliver positive signaling events downstream. For example, it activates PI3K–AKT and RAS signaling pathways to promote tumor cells proliferation and migration (50). Successful anti-PD-1/PD-L1 therapy requires adequate amount of specific T cells in tumor microenvironment and competent ability of T cells to get enough nutrients (57). Studies have shown aerobic glycolysis is essential for T cells to secrete IFN-γand attack tumor cells. PD-1/PD-L1 inhibitors may help T cells compete for glucose in tumor microenvironment, promoting T cell glycolysis and IFN-γ secretion (57, 58).

Daste et al. reported a case that a 64-years-old patient with HNSCC developed local tumor flare-up under immunotherapy, and a dramatic response was achieved in the following chemotherapy (59). Owing to the “loco-regional phenomena” described in their case study, they suggested that although clinical efficacy was not achieved in this case, immunotherapy might enhance response sensitivity to chemotherapy in patients with HNSCC (59).

### Overview of FDA-approved PD-1 inhibitors for head and neck cancers

#### Pembrolizumab

Pembrolizumab was the first anti-PD-1 antibody approved by FDA to treat patients with unresectable or metastatic melanoma who progress after Ipilimumab treatment. It is also approved for the treatment for melanoma patients harboring a *BRAF V600E* mutation, following treatment with a BRAF inhibitor. Pembrolizumab has also been legal for the treatment of non-small-cell lung cancer (NSCLC) without EGFR mutation and ALK rearrangement but with disease progression or following platinum-based chemotherapy (60). In August 2016, FDA approved the use of Pembrolizumab in R/M HNSCC that has progressed on or after platinum-containing chemotherapy (33, 34).

#### Nivolumab

Nivolumab, a PD-1 inhibitor, has been approved by FDA to treat Hodgkin lymphoma, renal cell carcinoma, NSCLC, and melanoma. Recent breakthrough in the application of Nivolumab in patients with processed HNSCC during chemotherapy or R/M HNSCC after chemotherapy with platinum-based drugs has made Nivolumab second to the Pembrolizumab approved by FDA in HNSCC treatment (35, 36).

## Clinical studies of PD-1/PD- L1 inhibitors

Inhibiting either PD-1 or PD-L1 function can block the PD-1 pathway. A number of PD-1/PD-L1 inhibitors are being investigated clinically and described in more details below (Table 2).

**Table 2:** Clinical Trials on anti-PD-1/PD-L1 in head and neck cancers.

**Immune checkpoint**	**Inhibitor**	**Other names**	**NCT-nummer**	**Phase**	**Arms**	**N of pts**	**Primary endpoint**	**Recruitment status**
PD-1	pembrolizumab	Lambrolizumab/MK-3475 /Keytruda	NCT02586207	Phase I	Pembrolizumab ^+^ Cisplatin ^+^ Radiation	58	AE	recruiting
			NCT02358031	Phase III	Pembrolizumab vs. Pembrolizumab^+^Platinum^+^5-FU vs. Cetuximab^+^Platinum^+^5-FU	825	PFS, OS	Active, not recruiting
			NCT02707588	Phase II	Pembrolizumab^+^radiotherapy vs. Cetuximab^+^radiotherapy	133	LRC	Active, not recruiting
	nivolumab	Opdivo/BMS-936558/MDX-1106/NIVO/ONO-4538	NCT02764593	Phase I	Nivolumab^+^Cisplatin vs. Nivolumab^+^High-dose Cisplatin vs. Nivolumab^+^Cetuximab vs. Nivolumab^+^IMRT	40	DLT	Active, not recruiting
			NCT03132038	Phase II	Nivolumab	92	non-progression rate	recruiting
			NCT03012581	Phase II	Nivolumab	300	ORR	recruiting
			NCT02105636	Phase III	Nivolumab vs. Cetuximab/Methotrexate/ Docetaxel	506	OS	Active, not recruiting
PD-L1	Durvalumab	Imfinzi/MEDI4736	NCT02207530	Phase II	Durvalumab	112	ORR	Active, not recruiting
			NCT02997332	Phase I	Durvalumab^+^Docetaxel^+^ Cisplatin^+^5-FU	36	RP2D, DLT	recruiting
			NCT02551159	Phase III	Durvalumab vs. Durvalumab^+^Tremelimumab vs. SOC	823	OS	Active, not recruiting
	Avelumab	Bavencio	NCT02952586	Phase III	Avelumab^+^SOC CRT vs. Placebo^+^SOC CRT	640	PFS	recruiting
			NCT02938273	Phase I	Avelumab^+^cetuximab^+^ Radiation therapy	10	toxicity	recruiting
	INCB024360		NCT02318277	Phase I/ II	MEDI4736 ^+^ INCB024360	42	DLT, AE, ORR	Active, not recruiting

*PD-L1, programmed death-1 ligand; FU, fluorouracil; HNSCC, head and neck squamous cell carcinoma; AE, adverse event; LRC, locoregional control; DLT, dose limiting toxicity; ORR, overall response rate; OS, overall survival; PFS, progression-free survival; SOC, standard of care; CRT, chemoradiation therapy; IMRT, intensity-modulated radiation therapy; RP2D, recommended phase II dose*.

### PD-1

#### Pembrolizumab (MK-3475, previously known as lambrolizumab)

Preclinical anti-tumor effects were demonstrated in animals bearing multiple tumors. The first phase I clinical trial was carried out in patients with advanced solid tumors (61). Results suggested that Pembrolizumab was well-tolerated and associated with durable antitumor activity in multiple solid tumors (61). Two mg/kg per 3 weeks is considered a safe and effective minimum dose of antitumor activity (61). KEYNOTE-012 trial was a multicenter, open-label, phase Ib trial that included patients with R/M HNSCC in one of the cohorts. The objective response rate (ORR) was ~20% and overall survival (OS) was better in HPV^+^ patients (33). Then a larger HNSCC expansion cohort of KEYNOTE-012 reported an ORR of 18.2%, and response rates were similar in HPV^+^ and HPV^−^ patients (62). In a recent single-arm, phase II KEYNOTE-055 study conducted in patients with R/M HNSCC, ORR was 16% and response rates were similar in HPV^+^ and HPV^−^ patients, providing rationale for treatment with Pembrolizumab (NCT02255097) (63).

Monotherapy with Pembrolizumab is being carried out in patients with NSCLC (NCT01840579), advanced solid tumors (NCT01295827) and hematologic malignancies (NCT01953692). Clinical trials of Pembrolizumab focusing on HNSCC are ongoing in comparison to chemotherapy (NCT02358031), in combination with radiotherapy (NCT02707588), and in combination with cisplatin and radiation (NCT02586207).

#### Nivolumab (MDX-1106, BMS-936558, ONO-4538)

The first phase I clinical trial was conducted in patients with treatment-refractory solid tumors such as advanced metastatic melanoma, colorectal cancer, castrate-resistant prostate cancer, NSCLC, and renal cell carcinoma (64). The study exhibited good tolerance and meaningful antitumor activity of PD-1 inhibitors, and the early results from a follow-up trial (NCT00730639) further confirmed this. It appeared that the PD-1 antibody was well-tolerated and demonstrated anti-tumor activity in many patients whose previous treatment failed (65). In a recent randomized, open-label, phase III clinical trial conducted in patients with R/M HNSCC, the ORR was 26.1% for Nivolumab, demonstrating a survival advantage compared with conventional treatments with ORR of 0% for investigators' choices of therapy (NCT02105636) (66). Ongoing clinical trials focusing on HNSCC include comparison to Cetuximab, Methotrexate or Docetaxel (NCT02105636), combination with Cisplatin, Cetuximab, or IMRT (NCT02764593), and monotherapy (NCT03132038, NCT03012581).

### PD-L1

#### Durvalumab (MEDI4736)

In a phase I/II clinical trial that included a group of HNSCC patients, ORR was 17%, especially higher (25%) in PD-L1^high^ patients. The disease control rate in PD-L1 high subgroup was 44.9%, much greater than that in PD-L1 low or negative subgroup (21.5%) (67). These data support continued clinical development of Durvalumab in HNSCC. Durvalumab is being tested as monotherapy (NCT02207530), in combination with Docetaxel plus Displatin and 5-FU (NCT02997332), and in comparison to Durvalumab plus Tremelimumab (NCT02551159).

#### Avelumab

Avelumab is an anti-PD-L1 antibody. Studies of Avelumab targeting HNSCC has been scarce. It's currently assessed in combination with Cetuximab and radiotherapy in a phase I trial (NCT02938273), and in combination with standard care in a phase III trial (NCT02952586).

## Factors influencing anti-PD-1/PD-L1 therapy

### Gut microbiota

It has been lately reported that gut microbiome plays important roles in many diseases, including influenza (68), multiple sclerosis (69, 70), diabetes (71), colorectal cancer (68, 72), and many others in various preclinical models, among which gut microbiome may modulate PD-1/PD-L1-based immunotherapy (73–76). Many kinds of bacteria have been proved to facilitate PD-1/PD-L1 blockades, meanwhile, there are bacteria that hamper the treatment (Table 3). It is reported that oral gavage of *Bifidobacterium* could achieve the same effects as anti-PD-L1 treatment, and combinational therapy almost eliminated tumor outgrowth, in which enhanced dendritic cell function led to more priming and accumulation of CD8^+^ T cells in the tumor microenvironment (76). On one hand, *Akkermansia muciniphila* was screened out to affect the anti-PD-1-based therapy in epithelial tumors in an IL-12 dependent fashion by enhancing the recruitment of CCR9^+^CXCR3^+^CD4^+^ T cells (75). Further study in patients also revealed that responding patients had more diverse and abundant bacteria of the *Ruminococcaceae* family, enhanced systemic and antitumor immunity, functioning better in anabolic pathways as well (74). On the other hand, the recent study by Matson V reported *Blautia obeum* and *Roseburia intestinalis* with compromised efficacy of PD-1 blockade (77). These results provide important information for cancer therapy with immune checkpoint inhibitors.

**Table 3:** Gut microbiome affecting efficacy of PD-1/PD-L1 treatment.

**Effects**	**Bacteria**	**Models**	**Other effects on immune systems**	**Author/year**	**References**
Enhanced efficacy	*Akkermansiacea muciniphila*	Human/mouse	Upregulating T_CM_, CD4/Foxp3 ratio in tumor sites and IL-12 production; Increasing IFN- γ production	Bertrand Routy 2018	(75)
	*Alistipes indistinctus*	Human/mouse	/	Bertrand Routy 2018	(75)
	*Bifidobacterium adolescentis*	Human	Decreasing peripherally derived Tregs	Matson V 2018	(77)
	*Bifidobacterium breve*	Mouse	Stimulating DCs directly, inducing DCs maturation and cytokine secretion	Ayelet Sivan 2015	(76)
	*Bifidobacterium longum*	Mouse	Promoting DCs maturation and inducing cytokine production	Ayelet Sivan 2015	(76)
	*Bifidobacterium longum*	Human	/	Matson V 2018	(77)
	*Collinsella aerofaciens*	Human	Decreasing peripherally derived Tregs	Matson V 2018	(77)
	*Enterococcus faecium*	Human	Decreasing peripherally derived Tregs	Matson V 2018	(77)
	*Enterococcus hirae*	Human/mouse	Upregulating T_CM_, CD4/Foxp3 ratio in tumor sites and IL-12 production; Increasing IFN- γ production	Bertrand Routy 2018	(75)
	*Klebsiella pneumonia*	Human	/	Matson V 2018	(77)
	*Parabacteroides merdae*	Human	Decreasing peripherally derived Tregs	Matson V 2018	(77)
	*Ruminococcaceae*	Human/mouse	Increasing effector T cells in peripheral blood and tumors	Gopalakrishnan V 2018	(74)
	*Veillonella parvula*	Human	/	Matson V 2018	(77)
Compromised efficacy	*Blautia obeum*	Human	/	Matson V 2018	(77)
	*Roseburia intestinalis*	Human	/	Matson V 2018	(77)

*T_CM_ central memory T cell; Treg regulatory T cell; DC dendritic cell*.

### Molecules regulating PD-1/PD- L1

Some tumors respond more sensitively to anti-PD-1/PD-L1 therapy, while others do not. The mechanisms regulating anti-PD-1/PD-L1 therapy sensitivity have arisen wide attention. Recently, two molecules, CMTM6 and CMTM4, have been reported as PD-L1 protein regulators. CMTM6 could prevent the degradation of PD-L1, maintaining the stability of PD-L1 and facilitating the immune escape of tumors. Interfering either CMTM6 or CMTM4 would hamper the expression of PD-L1. They function through reducing the ubiquitination of PD-L1, prolonging its half-life period. This provides a new target for immunotherapy to enhance the anti-PD-1/PD-L1 treatment (78, 79).

## Adverse events of FDA-approved PD-1 inhibitors and the relevant managements for head and neck cancers

The fact that PD-1/PD-L1 axis contributes to the maintenance of self-tolerance implies that immune checkpoint blockade might disturb the balance of immune systems, resulting in treatment-related adverse events (trAEs) (80) (Table 4). TrAEs are frequent and occur in up to 80% of patients treated with an PD-1/PD-L1 antibody (81, 82). In the KEYNOTE-012 trial and the KEYNOTE-055 trial, trAEs occured in 63%-65% HNSCC patients treated with Pembrolizumab (33, 63). The most commonly observed trAEs were fatigue, decreased appetite, rash, hypothyroidism, nausea and diarrhea (63). Grade 3–4 trAEs occurred in around 9–14% of patients who had PD-1 inhibitors treatment. Three deaths were reported due to pulmonary toxicity (53, 82).

**Table 4:** Incidents of treatment-related adverse events occurring in patients with head and neck cancers.

	**Pembrolizumab 10 mg/kg every 2 weeks Ib/n 60 (33)**		**Pembrolizumab 200 mg every 2 weeks Ib/n 132 (62)**		**Pembrolizumab 200 mg every 2 weeks II/n 171 (63)**		**Nivolumab 3 mg/kg every 2 weeks III/n 236(GLOBAL) (36)**		**Nivolumab 3 mg/kg every 2 weeks III/n 23(ASIAN) (66)**	
**Adverse Events**	**Grade 1–2**	**Grade 3–4**	**Grade 1–2**	**Grade 3–4**	**Any Grade**	**Grade 3–5**	**Any Grade**	**Grade 3–4**	**Any Grade**	**Grade 3–4**
Fatigue	20.00%	2.00%	21.00%	0	18.00%	1.00%	14.00%	2.10%	17.40%	0
Decreased appetite	0	0	7.00%	2.00%	5.00%	0	7.20%	0	21.70%	0
Rash	5.00%	2.00%	0	0	2.00%	1.00%	7.60%	0	17.40%	0
Nausea	0	0	5.00%	1.00%	6.00%	0	8.50%	0	8.70%	0
Hypothyroidism	0	0	11.00%	0	9.00%	0	0	0	0	0
Pruritus	12.00%	0	0	0	0	0	7.20%	0	17.40%	0
Diarrhea	2.00%	2.00%	0	0	6.00%	1.00%	6.80%	0	4.30%	0
Abdominal pain	0	0	1.00%	1.00%	0	0	0	0	0	0
Stomatitis	0	0	1.00%	1.00%	0	0	2.10%	0.40%	0	0
Colitis	0	0	0	1.00%	0	0	0	0	0	0
Lymphopenia	0	2.00%	0	0	0	0	0	0	0	0
Atrial fibrillation	0	2.00%	0	0	0	0	0	0	0	0
Congestive cardiac failure	0	2.00%	0	0	0	0	0	0	0	0
Neck abscess	0	2.00%	0	0	0	0	0	0	0	0
Alanine aminotransferase increase	0	3.00%	0	0	4.00%	0	0	0	0	0
Hyponatremia	0	3.00%	0	0	2.00%	1%%	0	0	0	0
Anemia	0	0	0	0	4.00%	2.00%	5.10%	1.30%	0	0
Musculoskeletal pain	2.00%	2.00%	0	0	0	0	1.30%	0	0	0
Immune thrombocytopenic purpura	0	0	0	1.00%	0	0	0	0	0	0
Dysphagia	0	0	1.00%	1.00%	0	0	0	0	0	0
Dehydration	0	0		1.00%	0	0	0	0	0	0
Facial swelling	0	0	2.00%	3.00%	0	0	0	0	0	0
Pneumonitis	0	0	2.00%	2.00%	4.00%	1.00%	0	0	0	0
Hyperglycemia	0	0	1.00%	1.00%	0	0	0	0	0	0
Asthenia	0	0	0	0	0	0	4.20%	0.40%	0	0

By comparing the various organs involved, grade 1–2 trAEs mainly influence the skin and the gut, while grade 3–4 events mainly affect the digestive tract. Data suggest that trAEs usually occur within 3–6 months after the PD-1/PD-L1 blockade treatment (83). Accumulative toxic effects with prolonged treatment of anti-PD-1 were not observed (65).

For T cell tumors, like T-cell non-Hodgkin's lymphoma (T-NHL), anti-PD-1/PD-L1 therapy could render the tumors better proliferative. The reason is in this kind of tumors, T cells don't play the role to attack the tumors, instead, they are the major part of the tumor. It highlights a dangerous possible adverse event of anti-PD-1 treatment (84).

### Nivolumab

A randomized, open-label, phase III study was designed to investigate efficacy and safety of Nivolumab for patients with recurrent HNSCC that progressed within 6 months post platinum-based chemotherapy (36). In this trial, the primary end point was OS. Although rates of trAEs of any grade were similar between two groups, fewer events of grade 3 or 4 were observed in the Nivolumab treatment group when treated with Nivolumab than the standard therapy group. Fatigue, nausea, rash, decreased appetite, and pruritus were the most commonly reported trAEs of any grade in patients receiving Nivolumab. Two treatment-related deaths owing to pneumonitis and hypercalcemia were reported in the Nivolumab treatment group (36). Daste et al. (59) reported a case of a patient with HNSCC developed tumor flare-up after therapy with Nivolumab (59).

### Pembrolizumab

TrAEs of any grade occurred within an average of 9 weeks after the initiation of Pembrolizumab (85, 86). In the KEYNOTE-012 trial, trAEs of any grade were observed in 63% of patients. The most frequently observed trAEs were fatigue, pruritus, nausea, decreased appetite and rash. Grade 3–4 trAEs were reported in 10 of 60 patients (17%), including increased ALT and AST, hyponatremia, atrial fibrillation and congestive heart failure (33). In the expansion cohort, 62% of patients had trAEs of any grade. The most common trAEs were fatigue, hypothyroidism and decreased appetite. Grade 3–4 trAEs were observed in around 9% of patients, including lowered appetite, facial swelling and pneumonitis (62). In the KEYNOTE-055 trial, around 64% of patients exhibited trAEs. Grade 3–5 trAEs were reported in 15% of patients. One death owing to treatment-related pneumonitis was reported (63).

### Severe immune-related adverse events in crucial organs

#### Myocarditis

Accounting for <0.3% of patients, myocarditis is a rare but severe immune-related adverse event that frequently results in rapid dyspnea and acute heart failure (87). More and more cases of patients with anti-PD-1/PD-L1 treatment-related heart diseases have been reported in recent 3 years (88). Semper et al. (89) reported a case of a patient, diagnosed with squamous cell carcinoma of the lung, developing Nivolumab-induced myocarditis. Three days post the 9th cycle of Nivolumab therapy, the patient with tumor remission developed acute chest pain and severe dyspnea, which was later confirmed to be immunotherapy-related (89). Johnson et al. (87) reported two more cases of patients, diagnosed with metastatic melanoma, developing lethal myocarditis induced by Nivolumab and Ipilimumab combined (87). Läubli et al. (90) reported a case of Pembrolizumab-induced myocarditis. A 73-years-old female patient with metastatic uveal melanoma developed severe Pembrolizumab-induced myocarditis which resulted in potentially life-threatening acute heart failure (90). In 2018, Frigeri et al. (91) reported the patients achieved complete remission of recurrent metastatic pulmonary adenocarcinoma after 7 cycles of Nivolumab administration. Unfortunately, she experienced rapid cardiogenic shock afterwards (91). A fatal case was reported by Matson et al. (92). One patient with NSCLC receiving Nivolumab developed acute heart failure (92). Moslehi et al. (88) have identified altogether 101 cases of severe immune checkpoint inhibitors-induced (ICIs-induced) myocarditis, 46% of which resulted in patients' deaths (88). A more conclusive mechanism of anti-PD-1-induced myocarditis is under investigation (87). Studies revealed that PD-L1 could be found on endothelium. Interaction between PD-1 and its ligands on endothelium is important in limiting T cell responses in the heart and thus controlling immune-mediated cardiac injury (93, 94). One suspected mechanism is that PD-L1 is expressed on the surface of various types of cells and tissues, including tumor cells and cardiac muscle cells. When patients receive anti-PD-1/PD-L1 treatment, owing to the distribution of drugs, T cell responses in cardiac muscles might be disturbed and enhanced, leading to the occurrence of lethal immune-related myocarditis (87, 95).

#### Pneumonitis

Incidence of pneumonitis of all grades during anti-PD-1 therapy was 2.7% and the incidence of pneumonitis for grade 3 or higher was around 0.8% (96). Patients diagnosed with lung cancers, compared to patients with other types of cancers had higher incidence of treatment-related pneumonitis, with incidence of grade 3 or higher being 1.8% and incidence of deaths being 0.4% (96, 97). In a randomized, open-label, phase II/III study on efficacy and safety of Pembrolizumab for patients with advanced NSCLC, three cases of deaths resulting from treatment-related pneumonitis were reported (85). As in clinical trial of PD-1 blockade treating HNSCC, two treatment-related deaths owing to pneumonitis and hypercalcemia were reported in the Nivolumab group of a randomized, open-label, phase III trial (NCT02105636) (36). In a phase II study, Bauml et al. evaluated efficacy of Pembrolizumab in patients with previously treated refractory head and neck cancers (KEYNOTE-055) and one death owing to immune-related pneumonitis was observed (63).

One patient with NSCLC, after receiving 2 cycles of anti–PD-1 therapy, developed symptoms of pneumonitis and received proper treatment. However, symptoms relapsed; treatments with corticosteroids displayed less efficacy and the patient died. Another case of a female patient with small-cell lung cancer (SCLC), treated with an anti-CTLA-4/PD-1 combination therapy, was reported. The patient showed responsiveness to corticosteroid treatment; with discontinuation of current immunotherapy, the patient recovered from pneumonitis and started next line of anti-tumor therapy (98).

#### Hepatitis

The incidence of immune-related hepatitis of all grades was around 3.1% and the incidence of grade 3 or higher was 0.5–0.6% (99). For a clinical trial with Pembrolizumab in patients with previously treated NSCLC (KEYNOTE-010), three cases of immune-related hepatitis were reported (97).

### Management of adverse events

**Figure 3:**
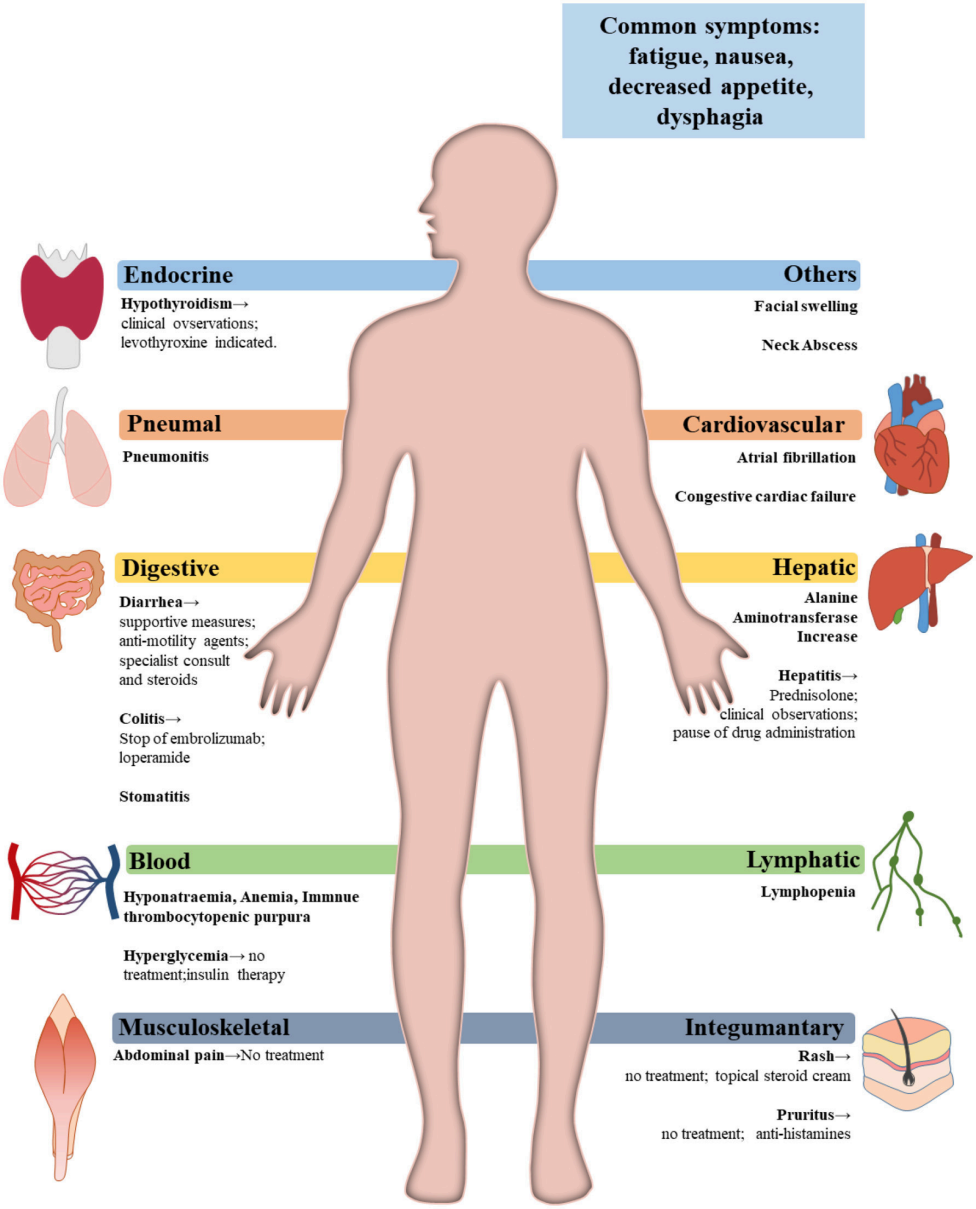
Main adverse events and treatments.

Before confirming the occurrence of immune-related adverse events, specialist should rule out all other possible diagnoses, including but not limited to infection and tumor progression (83). Figure 3 gives a glimpse of main adverse events in patients receiving anti-PD-1/PD-L1 therapy. The general principle for managing trAEs are suggested as followed: patients with grade 1 adverse events are provided with supportive care; patients with grade 2 events are advised on treatment with topical or systemic steroids (0.5–1 mg/kg/day); patients with grade 3 or 4 events require hospitalization, treatment of steroids, 1–2 mg/kg/day, or discontinuation of the current immunotherapy, depending on specialists' assessments (97, 100). Table 5 shows the management of some commonly observed trAEs. Most trAEs are manageable with steroids, which should be provided at a sufficient dose and gradually withdrawn. But there are some cases where trAEs may be permanent, and in those scenarios, adverse events can be treated with hormone instead (83, 100).

**Table 5:** Management of treatment-related rash, pneumonitis, thyroid dysfunction and diarrhea (100).

**Adverse events**	**Grade 1–2**	**Grade 3–4**
Rash	≤30% BSA: anti-histamines for pruritus and topical steroid cream for rash.	>30% BSA:skin biopsy is needed and steroids with 1 mg/kg of prednisolone until BSA≤30%.If life-threatening, permanently discontinue drug administration.
Pneumonitis	Clinical or diagnostic observations; delay drug administration; daily monitoring.	Oxygen is needed; stop drug administration; hospitalization; high dose steroids with methylprednisolone; intensive care support.
Thyroid dysfunction	Clinical or diagnostic observations; daily monitoring; for hypothyroidism, levothyroxine indicated; for hyperthyroidism, propranolol is needed.	Hospitalization; specialist consult; clinical observation
Diarrhea	≤6 bowel actions/day: supportive measures; anti-motility agents when needed.	>7 bowel actions/day: hospitalization; specialist consult; clinical observation; steroids with 1–2mg/kg prednisolone.

## The perspectives of anti-PD-1/PD-L1 therapy in head and neck cancers

**Figure 4:**
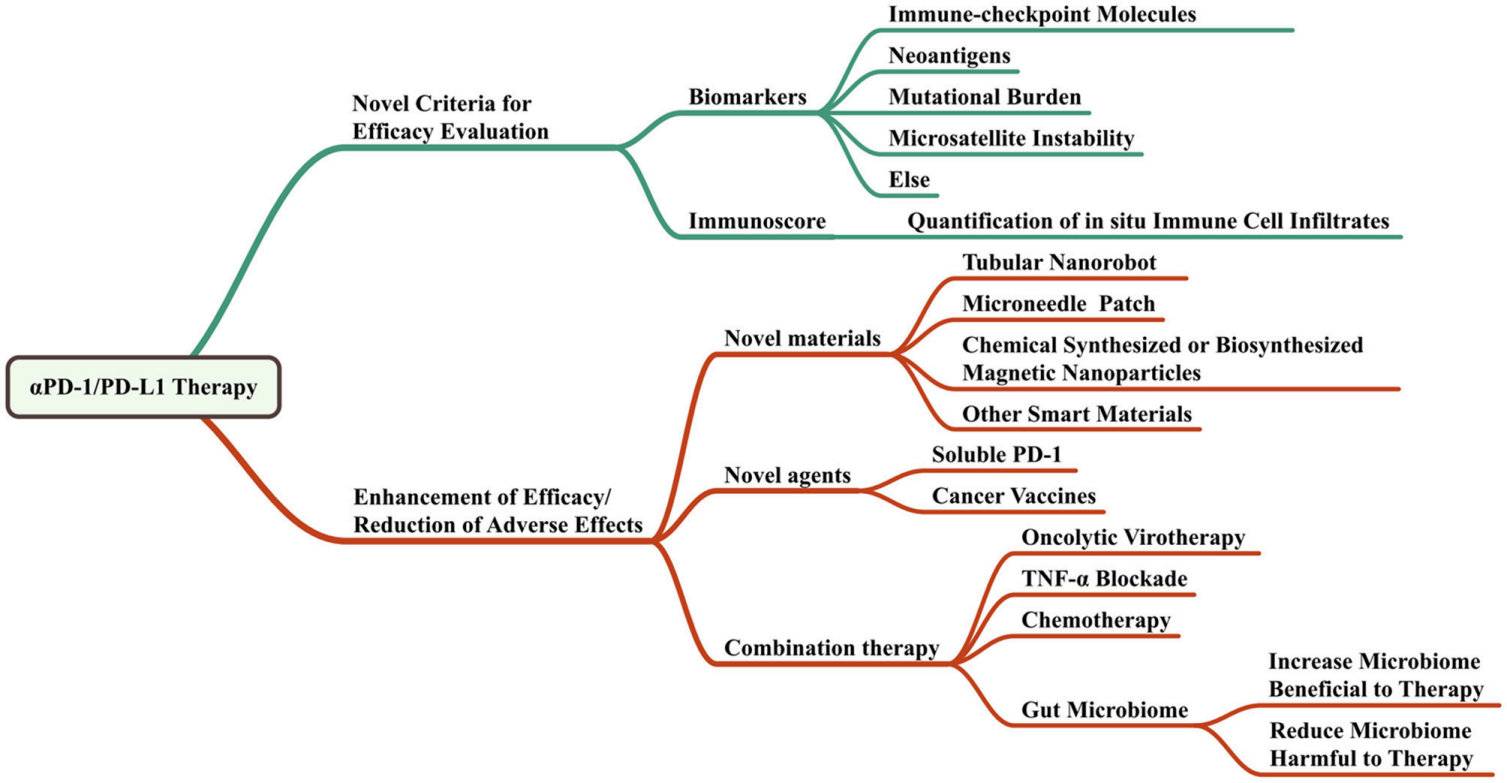
Perspectives of anti-PD-1/PD-L1 therapy.

Figure 4 shows the perspectives of anti-PD-1/PD-L1 therapy.

### Criteria to monitor the immune-checkpoint blockade

Scientists brought up the importance of monitoring immune-checkpoint blockade. As it is a novel therapy for cancers, the response evaluation and biomarkers should be different. Immune-related response criteria is an important concept to evaluate the immunotherapy and is the first step of precision immunotherapy (101). There are many biomarkers of immunotherapy response including PD-L1, other immune-checkpoint molecules, tumor-infiltrating lymphocytes (TILs), IFN-γ (102–104), mutational burden, neoantigens, microsatellite instability, serum markers, radiographic markers, and the “immunoscore” (105) which evaluates the distribution of TILs in the core and in the invasive margin of tumors. A recent study showed that the frequency of CD14^+^CD16^−^HLA^−^DR^hi^ monocytes had strong correlation with progression-free and OS in response to therapy with anti-PD-1. The researchers used single-cell mass cytometry to analyze the immune cell subpopulations in the peripheral blood of patients with stage IV melanoma before and after anti-PD-1 therapy. It is an effective predictive biomarkers of a clinical response (106). Similarly, more predictive biomarkers are expected to be found and used in the near future.

### Novel materials advancing the effect

Nanoscale materials have potential as drug delivery systems that assist or advance the treatment in cancers. Some could even respond intelligently to molecular triggers (107, 108). A recent research reported that an autonomous DNA robot was programmed to transport blood coagulation protease thrombin within tubular nanorobot while DNA outside of the nanorobot as both a targeting domain and a molecular trigger. It could target the nucleolin specifically expressed in tumor blood vessels and caused tumor necrosis. Animal experiments with this DNA robot showed promising results (109). As it could carry the blood coagulation protease thrombin that is a type of protein, it would also be able to transport the anti-PD-1/PD-L1 antibody to specific areas with certain DNA targeting domains.

A microneedle, made by hyaluronic acid and pH-sensitive dextran nanoparticles, is developed to encapsulate anti-PD-1 antibody and glucose oxidase. Glucose oxidase can turn blood glucose into gluconic acid and generate an acidic environment in tumors to drive the self-dissociation of nanoparticles and finally substantially release anti-PD-1 antibodies. This newly developed tool with immunotherapy induced more robust immune response in melanoma. And the microneedle could carry more than one antitumor therapeutics like combination of anti-PD-1 and anti-CTLA-4 antibodies to enhance the treatment effect (110).

Years ago, Sun et al. utilized bacterial magnetosomes as drug carriers transporting doxorubicin to treat hepatocellular carcinoma and got a better result compared with the sole doxorubicin group (111). Immobilization of anti-PD-1/PD-L1 antibodies on magnetic nanoparticles may also provide an efficient local delivery strategy of the drugs for malignant solid tumors. Local magnetic delivery of these immobilized antibodies would increase local concentration while reduce the administration times, total usage and peripheral distribution of the antibodies, reducing the adverse effects. It would be very easy to immobilize antibodies on either biosynthesized or chemical synthesized magnetic nanoparticles since there are a lot of linking methods available (112).

### Novel agents providing similar blockade effects of anti-PD-1/PD-L1 antibodies

Despite the anti-PD-1/PD-L1 antibodies, soluble PD-1 (sPD-1) peptides may provide similar inhibition effect of PD-1 pathway by competitively binding to PD-L1 expressed on tumor cells. The plasmids expressing sPD-1 peptides could also be developed as gene therapy drugs which turn tumor cells as producers of sPD-1.

#### Soluble immune checkpoint molecules

In addition to membrane bound form, there are sPD-1 and soluble PD-L1 (sPD-L1). Currently, sPD-1 is thought to be the translational product of the PD-1Δex3 mRNA transcript, and sPD-L1 may be derived from the cleavage of membrane bound PD-L1 by matrix metalloproteinases.

sPD-1 and sPD-L1 can also bind to ligands, thus blocking the PD-1/PD-L1 signaling pathway, resulting in potent peripheral T-cell anti-tumor responses. It's reported that the PD-1 extracellular domain was transfected into tumors by adenoviral vectors and could antagonize the negative regulation of T cells by PD-1/PD-L1 pathway, thus inhibiting tumor growth and prolong survival of mice (113).

Compared with membranous molecules, soluble molecules can not only affect neighboring cells in the tumor microenvironment, but also affect the body farther through the blood circulation, having a wider range of biological effects.

The production and function of the sPD-1 and sPD-L1 require further investigation. sPD-1 and sPD-L1 can be used in immunomodulatory therapy in combination with other antitumor therapy, such as HSP70 vaccine, to enhance the anti-tumor efficacy of tumor vaccine (114). In addition, the soluble forms may be used as an additional biomarker to the membrane bound forms, helping more accurately determine the patient's immune status and predict efficacy (115).

#### Cancer vaccines

Up to now, preclinical and recent clinical studies have indicated that combining PD-1 or PD-L1 checkpoint inhibitors with cancer vaccines improves antitumor activity compared with anti-PD-1 or PD-L1 antibody monotherapy alone (116). However, satisfactory results about vaccines targeting PD-1 or PD-L1 checkpoint molecular are few. The DNA vaccines under active study work well but safety is hard to guarantee. In contrast, protein vaccines are low in cost and high in safety. It provides a promising research direction for the future development of cancer treatment. A study using genetic engineering to prepare a Cholera Toxin B based vaccine that targets both mouse MUC1 and mouse PD-1 showed that this fused protein vaccine can produce a stronger immune response (117).

### Combination therapy

Luo et al. (118) developed a nano-vaccine by simply mixing an antigen with a synthesized polymeric nanoparticle, PC7A NP. It delivered tumor antigens to APCs in draining lymph nodes, increasing surface presentation and simultaneously activating type I interferon-triggered genes through STING pathway. Combination of PC7A nano-vaccine with anti-PD-1 antibodies demonstrated increased survival rate in animal tumor models. Tumor growth was completely inhibited when these vaccinated animals were rechallenged with tumor cells, suggesting generation of antitumor memory (118). Researchers found that exploiting the individual tumor mutations as neo-epitopes and utilizing them as vaccines could enhance the immune response to tumors. Some patients even completely responsed to vaccination during combinational therapy with anti-PD-1 (119, 120).

Oncolytic virotherapy has demonstrated promise, however, it only had efficacy in a small fraction of tumor patients. As the virus could upregulate PD-L1 expression on tumor cells, combination of oncolytic virus, and anti-PD-1/PD-L1 therapy could synergistically promote the treatment of cancers. This was tested in colon and ovarian cancer models, but was believed to own wider indications (121).

Recent study revealed that TNF-α blockade prevents death of tumor infiltrating T lymphocyte induced by anti-PD-1 as well as PD-L1 and TIM-3 expression. It is strongly rationalized to develop a combinational therapy with anti-PD-1/PD-L1 and anti-TNF-α in cancer patients (122).

Chemotherapy drug gemcitabine (GEM) and anti-PD-L1 antibodies could be released locally when an engineered reactive oxygen species (ROS)-degradable hydrogel was injected and formed in tumor microenvironment, which contained abundant ROS. Anti-PD-L1-GEM scaffold promoted an tumor regression in the tumor-bearing mice and prevention of tumor recurrence after primary resection (123). In this research, a novel material together with the combination therapy reinforced the effect and reduced side effects of the treatment.

The trends of anti-PD-1/PD-L1 therapy are to enhance the therapy effects while reduce the side effects. It would benefit from the combination of anti-PD-1/PD-L1 antibodies with other checkpoint inhibitors, other suppressor inhibitors, cytokine inhibitors or chemotherapy drugs. Emerging novel materials and delivery strategies like nanorobots, microneedle patches, and magnetic immobilization could help the therapeutics work better in the way of localizing them in the cancer sites or carrying other biomarkers like DNAs or proteins to target better.

## Author contributions

BY, TL, YQ, and HL summarized the literature, wrote the manuscript, and prepared figures. SZ and BC provided critical comments and wrote part of the manuscript. JS supervised all the work and wrote the manuscript.

### Conflict of interest statement

The authors declare that the research was conducted in the absence of any commercial or financial relationships that could be construed as a potential conflict of interest.

## Abbreviations

APC: antigen presenting cell
ATF: activating transcription factor
CRC: colorectal cancer
GEM: chemotherapy drug gemcitabine
GOx: glucose oxidase
HNSCC: head and neck squamous cell carcinoma
IGF: insulin-like growth factor
NFAT: nuclear factor of activated T cells
NSCLC: non-small cell lung cancer
ORR: objective response rate
OS: overall survival
PIP: phosphatidylinositol
PLGF: placental growth factor
RCC: renal cell carcinoma
ROS: reactive oxygen species
RTK: receptor tyrosine kinases
SAEs: severe adverse events
sPD-1/sPD-L1: soluble PD-1/ soluble PD-L1
TCR: T cell receptors
TGF: transforming growth factor
TILs: tumor-infiltrating lymphocytes
TKIs: tyrosine kinase inhibitors
TNF: tumor necrosis factor
T-NHL: T-cell non-Hodgkin's lymphoma
trAEs: treatment-related adverse events.
